# Comparison of long-term outcomes of complete vs. incomplete revascularization in elderly patients (≥75 years) with acute coronary syndrome and multi-vessel disease undergoing percutaneous coronary intervention

**DOI:** 10.3389/fcvm.2023.1037392

**Published:** 2023-07-25

**Authors:** Yu-Ying Lu, Chen-Hung Lee, Chun-Chi Chen, Dong-Yi Chen, Ming-Yun Ho, Jih-Kai Yeh, Yu-Chang Huang, Chieh-Yu Chang, Chao-Yung Wang, Shang-Hung Chang, I-Chang Hsieh, Ming-Jer Hsieh

**Affiliations:** ^1^ Division of Cardiology, Department of Internal Medicine, Chang Gung Memorial Hospital, Chang Gung University College of Medicine, Taoyuan, Taiwan; ^2^ College of Medicine, Chang Gung University, Taoyuan, Taiwan; ^3^ Center for Big Data Analytics and Statistics, Department of Medical Research and Development, Chang Gung Memorial Hospital, Linkou, Taiwan

**Keywords:** elderly, complete revascularization (CR), acute coronary syndrome (ACS), multi-vessel disease, percutaneous coronary intervention

## Abstract

**Background:**

The optimal revascularization strategy for elderly patients with acute coronary syndrome (ACS) remains uncertain. We evaluated the impact of complete revascularization (CR) vs. incomplete revascularization (IR) in elderly ACS patients with multivessel disease (MVD) undergoing percutaneous coronary intervention (PCI).

**Methods:**

Using registry data from 2011 to 2019, we conducted a propensity-score matched cohort study. Elderly patients (≥75 years) with ACS and MVD who underwent PCI were divided into CR and IR groups based on angiography during index hospitalization. Major adverse cardiovascular events (MACEs), including all-cause mortality, recurrent non-fatal myocardial infarction, and any revascularization, were assessed at 3-year follow-up.

**Results:**

Among 1,018 enrolled patients, 496 (48.7%) underwent CR and 522 (51.3%) received IR. After 1:1 propensity-score matching, we analyzed 395 pairs. At 3-year follow-up, CR was significantly associated with lower MACE risk compared to IR (16.7% vs. 25.6%, HR = 0.65, 95% CI: 0.47–0.88, *p* = 0.006), driven by reduced all-cause mortality. This benefit was consistent across all pre-specified subgroups, particularly in ST segment elevation (STE)-ACS patients. In non-STE (NSTE)-ACS subgroup analysis, CR was also associated with a lower risk of cardiac mortality compared to IR (HR = 0.30, 95% CI: 0.12–0.75, *p* = 0.01).

**Conclusion:**

In elderly ACS patients with MVD undergoing PCI, CR demonstrates superior long-term outcomes compared to IR, irrespective of STE- or NSTE-ACS presentation.

## 1. Introduction

Early invasive therapy rather than conservative therapy is recommended for high-risk patients presenting with acute coronary syndrome (ACS) (1). With regards to the revascularization strategy for multi-vessel disease (MVD), compared to culprit-only or incomplete revascularization (IR), complete revascularization (CR) is recommended due to better long-term survival in patients with ACS (1, 2). In the recent decade, about 30% of ACS occurs in patients ≥75 years of age and the incidence of ACS in elderly people is also expected to rise with the increasing life expectancy (3, 4). However, the superiority of CR over IR in elderly patients with ACS and MVD is still under debate.

Older patients aged ≥75 years have often been excluded or only constituted a small proportion of research subjects in previous revascularization studies (2). In addition, age is a well-known risk factor for cardiovascular events, and its interaction with revascularization therapy is also complicated. The DANAMI-3-PRIMULTI randomized trial found that the benefit of CR after primary percutaneous coronary interventions (PCI) was attenuated with increasing age (5). Older patients are more likely to have higher rates of comorbidities such as hypertension, diabetes mellitus, and chronic kidney disease (CKD), and more likely to have complex coronary lesions and abnormal heart function, all of which both hinder the achievement of CR and also have a great impact on clinical results (6). Thus, in real-world clinical scenarios, elderly patients with ACS often receive conservative therapy or IR rather than CR. Randomized control trials addressing this issue targeted at elderly patients are still lacking. Hence, the aim of this retrospective propensity score-matched study is to compare 3-year clinical outcomes between IR and CR in elderly adults (≥75 years) with ACS and MVD undergoing PCI.

## 2. Materials and methods

### 2.1. Subjects and study design

**Figure 1:**
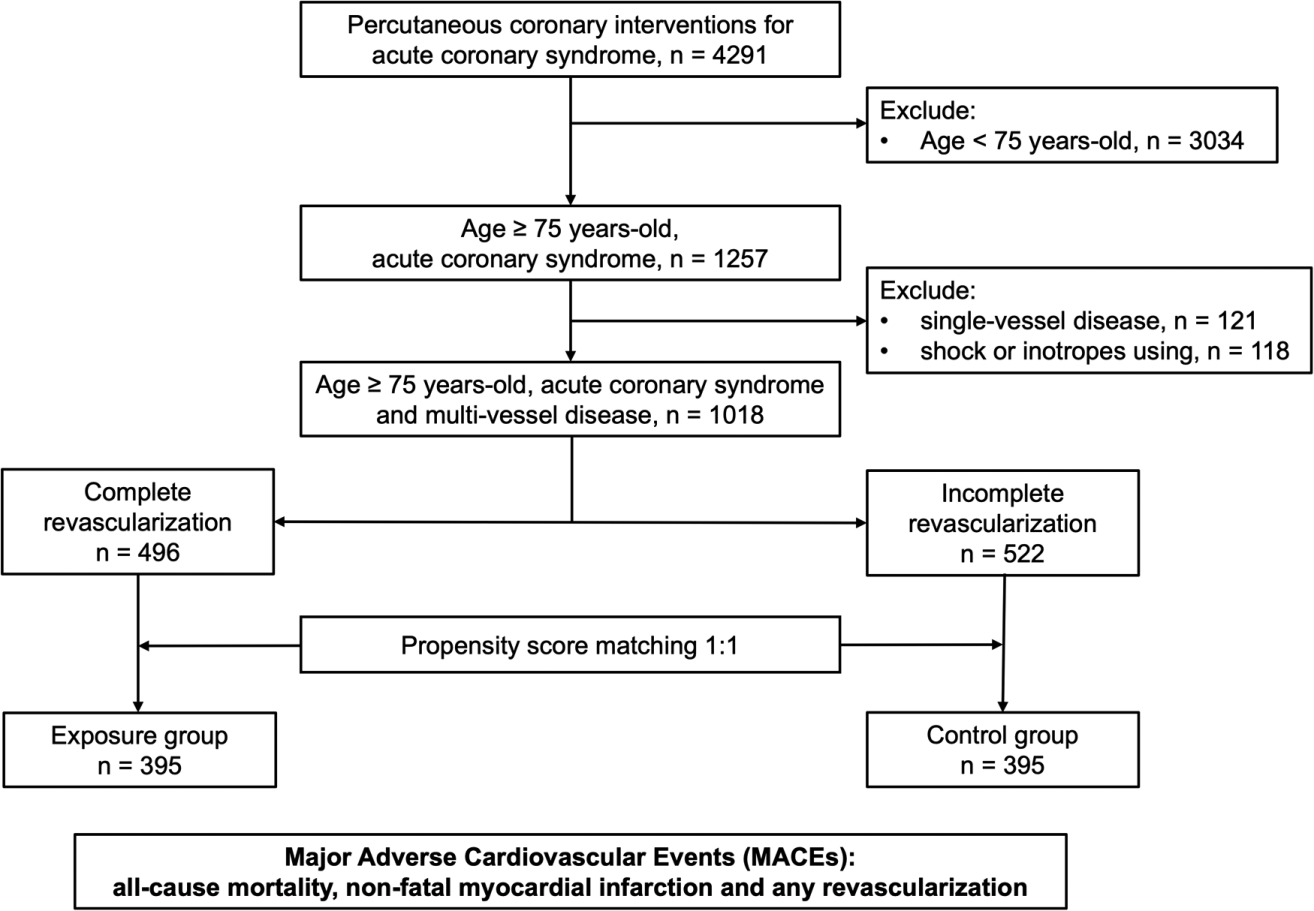
Flowchart of patient enrollment.

From April 2011 to May 2019, 4,291 patients with ACS who received percutaneous coronary interventions at our department were included for further analysis. Patients aged <75 years (*n* = 3,034) and those aged ≥75 years with single vessel disease (*n* = 121), shock or inotropes using (*n* = 118) were excluded. The patients were further divided into either IR or CR groups according to the final angiography results at the index hospitalization. The study flow chart was showed in Figure 1. The date of percutaneous coronary interventions completion was defined as the first day of enrolment. All patients were followed up for 3–6 months at outpatient clinics or by phone until completing 3 years of follow-up. Major adverse cardiovascular events (MACEs) were a composite endpoint including all-cause mortality, recurrent non-fatal myocardial infarction, and any revascularization (either percutaneous coronary interventions or bypass surgery) after 3 years of follow-up. All patients signed informed consent for clinical registry participation after the percutaneous coronary interventions, and the study was approved by the local Institutional Review Board (No. 201101154B0).

### 2.2. Definitions

Old age in this study was defined as an age ≥75 years. ACS, including both ST segment elevation (STE)- and non-ST segment elevation (NSTE)-ACS, was defined as chest pain with one or more of the following: (1) EKG with STE in two contiguous leads with or without reciprocal ST segment depression; (2) elevated biomarkers of myocardial necrosis including troponin-I or CK-MB; and (3) EKG with ST segment depression or inverted T wave in two contiguous leads. MVD was defined as >50% stenosis in ≥2 epicardial coronary arteries that were ≥2.5 mm in diameter in angiography. CR was defined as the absence of ≥50% stenosis in major epicardial coronary arteries or their side branches with a diameter ≥2.5 mm after successful PCI during the index hospitalization. The patients who did not meet the CR criteria were defined as having IR. This was a retrospective clinical observational study, and all strategies were decided by clinical physicians according to patient's individual condition. All the CR in this study were achieved in the index hospitalization. There was no scheduled staged revascularization after the index hospitalization.

### 2.3. Statistical analysis

Differences between the IR and CR groups in baseline characteristics, comorbidities, presentations, left ventricular ejection fraction (LVEF), and baseline coronary anatomy were assessed. The continuous variables in our dataset had normal distribution and were thus summarized as mean (± standard deviation) and compared using the *t*-test. Categorical variables were expressed as percentage and compared using the *χ*^2^ or Fisher's exact tests.

To account for confounding, potential clinical covariates were introduced to construct the propensity score with 1:1 matching. The revascularization strategy (CR or IR) was set as a dependent variable, whereas parameters that were clinically relevant for the selection of CR or IR, including age, sex, hypertension, hyperlipidemia, diabetes mellitus, LVEF, prior stroke, prior myocardial infarction, CKD stage, presentation of ACS (STE-ACS or NSTE-ACS), Killip class, chronic total occlusion, calcification, and bifurcation were set as independent variables. To determine an appropriate sample size, we performed power analysis using the estimated incidences of MACEs in IR as 25% and CR as 15% based on previous study results (7). The required sample size was 668 patients (334 patients in each group) assuming a statistical power (1-β) of 90% and a sensitivity (α) of 5%. Therefore, we performed propensity score matching using the more liberal match tolerance that was set as a width of 0.30 multiplied by the standard deviation of the propensity score distribution, and the generated sample size was 790 patients (395 patients in each group).

A Cox proportional hazards model was used to estimate interactions and relative risks of endpoints between the IR and CR groups. Cumulative MACE rates were also presented as Kaplan–Meier curves with log-rank tests. Subgroup analysis was conducted to determine whether the hazard ratio (HR) of MACEs in the CR and IR groups were similar in the pre-specified subgroups, including age, sex, diabetes mellitus, hypertension, LVEF < 40%, Killip class 3, CKD stage ≥3, ACS presentation type, and calcified coronary lesion. Additional subgroup analysis focus on patients with NSTE-ACS were provided. All statistical analyses were performed using IBM-SPSS (Version 24, Chicago, Illinois, USA) statistical software. Statistical significance was established at a 2-sided *p* < 0.05 for all tests.

## 3. Results

### 3.1. Patient characteristics

A total of 1,018 elderly patients with ACS and MVD were enrolled, of whom 522 were classified into the IR (51.3%) group and 496 were classified into the CR (48.7%) group. Propensity score matching was performed to adjust bias between the IR and CR groups in baseline clinical characteristics. The baseline clinical characteristics of the two groups before and after propensity score matching were compared and are shown in Table 1. Before matching, the IR group were older (80.1 ± 4.1 vs. 79.4 ± 3.7 years, *p* = 0.007), had a lower LVEF (55.0% ± 14.4% vs. 58.5% ± 14.4%, *p* < 0.001), and had more STE-ACS (31.4% vs. 24.0%, *p* = 0.01) than the CR group. No significant differences were observed in sex, diabetes mellitus, hypertension, hyperlipidemia, smoking, CKD stage ≥3, previous stroke, chronic obstructive pulmonary disease, severe liver disease, malignancy, prior myocardial infarction, clinical frailty scale, or complex coronary anatomy, including calcified lesions, bifurcation lesions, chronic total occlusion, type B2/C lesions (8). After 1:1 propensity score matching, 395 pairs of patients were included in each group, and there were no significant differences in any of the characteristics listed in Table 1 between the two groups.

**Table 1:** Baseline characteristics of elderly patients with MI and multivessel disease before and after propensity score matching.

	Before propensity score matching			After propensity score matching		
	IR	CR	*p*-value	IR	CR	*p*-value
Patient number, *n*	522	496		395	395	
Age, years old	80.1 ± 4.1	79.4 ± 3.7	0.007	79.5 ± 3.8	79.6 ± 3.8	0.640
Female gender, *n* (%)	162 (31.0)	162 (32.7)	0.591	118 (29.9)	124 (31.4)	0.700
Diabetes mellitus, *n* (%)	226 (43.3)	202 (40.7)	0.410	157 (39.7)	160 (40.5)	0.885
Hypertension, *n* (%)	371 (71.1)	350 (70.6)	0.890	278 (70.4)	286 (72.4)	0.582
Hyperlipidaemia, *n* (%)	172 (33.0)	153 (30.8)	0.501	139 (35.2)	128 (32.4)	0.452
Smoking, *n* (%)	118 (22.6)	92 (18.5)	0.121	96 (24.3)	77 (19.5)	0.121
CKD stage ≥3, *n* (%)	161 (30.8)	127 (25.6)	0.070	91 (23.0)	106 (26.8)	0.250
Previous stroke, *n* (%)	51 (9.8)	39 (7.9)	0.321	34 (8.6)	33 (8.4)	1.000
COPD, *n* (%)	51 (9.8)	65 (13.1)	0.114	27 (6.8)	40 (10.1)	0.125
Severe liver disease, *n* (%)	5 (1.0)	7 (1.4)	0.570	2 (0.5)	3 (0.8)	1.000
Malignancy, *n* (%)	57 (10.9)	60 (12.1)	0.557	44 (11.1)	48 (12.2)	0.739
Prior MI, *n* (%)	50 (9.6)	66 (13.3)	0.075	44 (11.1)	50 (12.7)	0.583
LVEF, mean (%)	55.0 ± 14.4	58.5 ± 14.4	<0.001	55.7 ± 13.8	57.3 ± 14.5	0.123
LVEF < 40%, *n* (%)	85 (16.3)	66 (13.3)	0.187	55 (13.9)	47 (11.9)	0.458
Clinical presentation			0.010			0.180
NSTE-ACS, *n* (%)	358 (68.6)	377 (76.0)		274 (69.4)	292 (73.9)	
STE-ACS, *n* (%)	164 (31.4)	119 (24.0)		121 (30.6)	103 (26.1)	
Killip Class ≥3, *n* (%)	113 (21.6)	58 (11.7)	<0.001	63 (15.9)	54 (13.7)	0.423
Calcified lesion, *n* (%)	178 (34.1)	160 (32.3)	0.549	133 (33.7)	139 (35.2)	0.708
Bifurcation lesion, *n* (%)	39 (7.5)	46 (9.3)	0.310	29 (7.3)	26 (6.6)	0.780
Chronic total occlusion, *n* (%)	57 (10.9)	59 (11.9)	0.693	45 (11.4)	49 (12.4)	0.742
B2/C type lesion, *n* (%)	459 (87.9)	448 (90.3)	0.229	343 (86.8)	354 (89.6)	0.270
Clinical frailty scale, average	4.0 ± 1.3	4.1 ± 1.5	0.348	4.0 ± 1.5	4.1 ± 1.6	0.278
Clinical frailty scale ≥5, *n* (%)	119 (22.8)	127 (25.6)	0.306	87 (22.0)	103 (26.1)	0.212

CKD, chronic kidney disease; COPD, chronic obstructive pulmonary disease, LVEF, left ventricular ejection fraction; MI, myocardial infarction; NSTE-ACS, Non-ST segment elevation acute coronary syndrome; STE-ACS, ST segment elevation acute coronary syndrome.

### 3.2. Clinical outcomes

**Figure 2:**
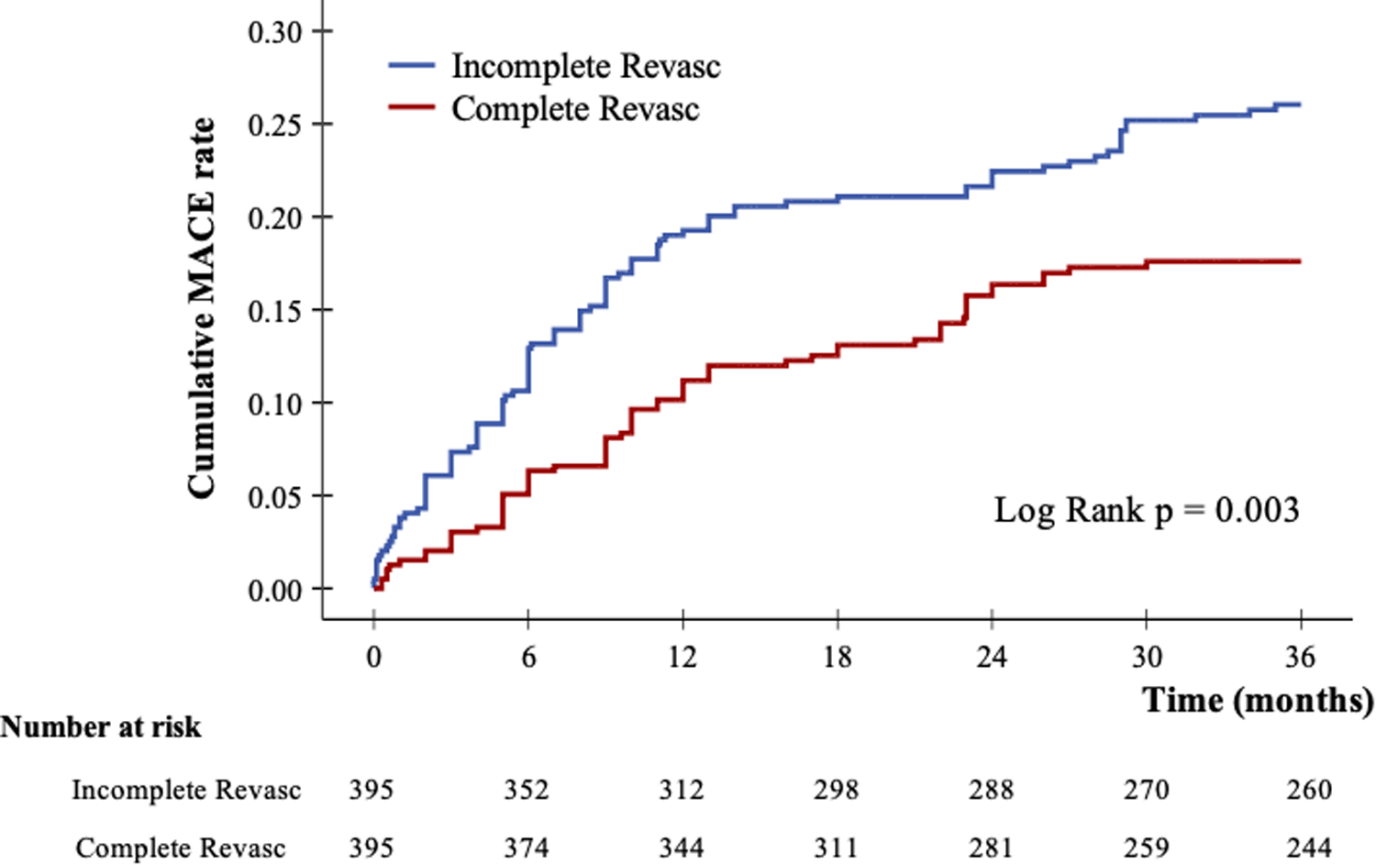
Kaplan–Meier curves of cumulative incidence of major adverse cardiovascular events after 3-year follow up.

After 3 years of follow-up, 167 (21.1%) patients developed MACEs. Table 2 shows the clinical outcomes and relative risks between the IR and CR groups after 3 years of follow-up. The incidence rates of MACEs were 16.7% in the CR group and 25.6% in the IR group. The incidence rates of all-cause mortality per 1,000 patient-years were 65.1 and 103.3 in the CR and IR groups, respectively. Compared with the IR group, the CR group had a significantly lower risk of all-cause mortality in the Cox proportional hazards model (HR: 0.65; 95% CI: 0.47–0.88, *p* = 0.006). The Kaplan–Meier survival curves for cumulative MACEs between the CR and IR groups are displayed in Figure 2 (log-rank *p* = 0.003).

**Table 2:** Three-year follow-up clinical outcomes and relative risks between IR and CR.

Clinical outcomes	Patient number, *n*	Event number, *n*	Event/patient number, %	Incidence per 1,000 person-years	Hazard ratio	95% confidence interval	*p*-value
All-cause mortality							
IR	395	71	18.0	68.8	1.00	[Reference]	
CR	395	30	7.6	26.7	0.38	0.24–0.62	<0.001
Non-fatal MI							
IR	395	12	3.0	11.4	1.00	[Reference]	–
CR	395	9	2.3	8.0	0.71	0.30–1.69	0.444
Any revascularization							
IR	395	47	11.9	45.8	1.00	[Reference]	–
CR	395	43	10.9	38.2	0.82	0.54–1.24	0.341
MACEs							
IR	395	101	25.6	103.3	1.00	[Reference]	–
CR	395	66	16.7	65.1	0.65	0.47–0.88	0.006

CR, complete revascularization; IR, incomplete revascularization; MACEs, major adverse cardiovascular events; MI, myocardial infarction.

The incidence rates of all-cause mortality at 3 years of follow-up were 7.6% in the CR group and 18.0% in the IR group. The incidence rates of all-cause death per 1,000 patient-years were 26.7 and 68.8 in the CR and IR groups, respectively. Compared with the IR group, the CR group had a significantly lower risk of all-cause mortality in the Cox proportional hazards model (HR: 0.38; 95% CI: 0.24–0.62, *p* < 0.001).

The incidence rates of recurrent non-fatal myocardial infarction were 2.3% in the CR group and 3.0% in the IR group. The incidence rates of any revascularization were 10.9% in the CR group and 11.9% in the IR group. There were no statistically significant differences in non-fatal myocardial infarction (*p* = 0.444) and any revascularization (*p* = 0.341) between the two groups.

### 3.3. Subgroup analysis

**Figure 3:**
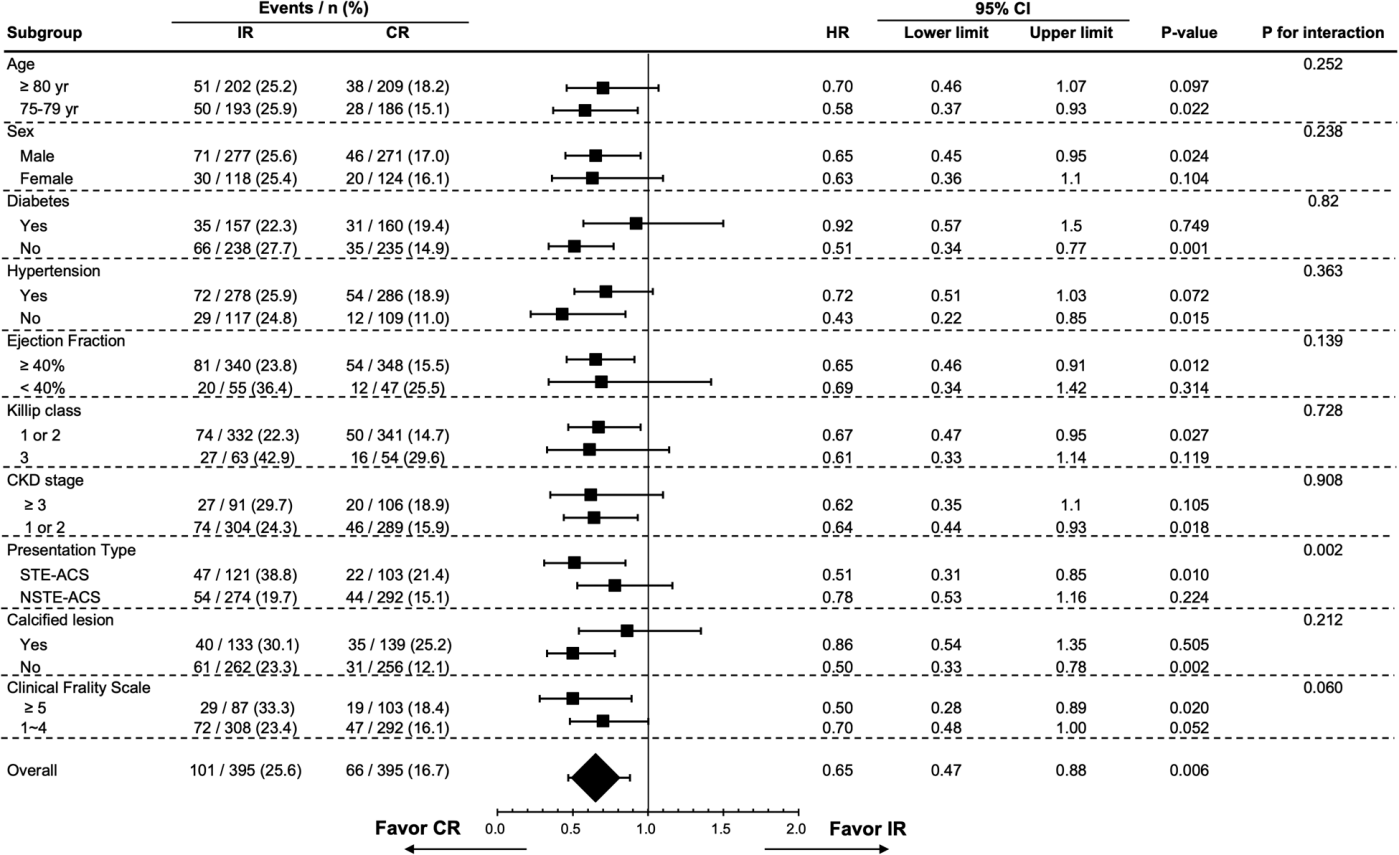
Subgroup analysis of 3-year major adverse cardiovascular events by selected baseline characteristics in the propensity score matched cohort. CI, confidence interval; CKD, chronic kidney disease; CR, complete revascularization; HR, hazard ratio; IR, incomplete revascularization; NSTE-ACS, Non-ST segment elevation acute coronary syndrome; STE-ACS: ST-segment elevation acute coronary syndrome.

Figure 3 shows the results of subgroup analysis for the occurrence of MACEs according to baseline characteristics in the matched study population. In general, the trend of a better reduction in the risk of MACEs in the CR group than in the IR group was consistent across all pre-specified subgroups. Age ≥80 years (*p* = 0.252), sex (*p* = 0.238), hypertension (*p* = 0.363), diabetes mellitus (*p* = 0.820), LVEF < 40% (*p* = 0.139), Killip class 3 (*p* = 0.728), CKD stage ≥3 (*p* = 0.908), calcified coronary lesions (*p* = 0.212) and clinical frailty scale (*p* = 0.06) did not modify the treatment effect. A nominally significant interaction between ACS presentation type (STE-ACS or NSTE-ACS) and the treatment effect on MACEs was found (*p* = 0.002). Although both HRs in STE-ACS and NSTE-ACS were less than 1.0, interaction analysis showed a greater benefit regarding MACEs in the CR group than in the IR group with STE-ACS.

### 3.4. Subgroup analysis for patients with NSTE-ACS

We conducted further analysis on patients with NSTE-ACS in the PSM cohort. The baseline characteristics, comorbidity, and clinical frailty scale between the IR and CR groups were not significantly different, as shown in Table 3. Table 4 displays the clinical outcomes and relative risks between the IR and CR groups in NSTE-ACS patients after 3 years of follow-up. The risks of non-fatal myocardial infarction (HR: 0.90; 95% CI: 0.29–2.80, *p* = 0.861), any revascularization (HR: 0.97; 95% CI: 0.59–1.60, *p* = 0.898), and MACEs (HR: 0.78; 95% CI: 0.53–1.16, *p* = 0.224) were not significantly different between the IR and CR groups in NSTE-ACS. However, CR in NSTE-ACS patients had a significantly lower risk of all-cause mortality than IR (HR: 0.39; 95% CI: 0.22–0.70, *p* = 0.002). This effect was mainly driven by a reduction in cardiac mortality (HR: 0.30; 95% CI: 0.12–0.75, *p* = 0.010), rather than non-cardiac mortality (HR: 0.47; 95% CI: 0.22–1.01, *p* = 0.053).

**Table 3:** Comparisons of baseline characteristics between IR and CR in NSTE-ACS in PSM cohort.

	NSTE-ACS		
	IR	CR	*p-*value
Patient number, *n*	274	292	
Age, years old	79.4 ± 3.6	79.8 ± 3.8	0.178
Female gender, *n* (%)	89 (32.5)	100 (34.2)	0.721
Diabetes mellitus, *n* (%)	120 (43.8)	127 (43.5)	1.000
Hypertension, *n* (%)	201 (73.4)	215 (73.6)	1.000
Hyperlipidaemia, *n* (%)	108 (39.4)	101 (34.6)	0.257
Smoking, *n* (%)	56 (20.4)	55 (18.8)	0.672
CKD stage ≥3, *n* (%)	70 (25.5)	81 (27.7)	0.570
COPD, *n* (%)	14 (5.1)	26 (8.9)	0.100
Severe liver disease, *n* (%)	1 (0.4)	1 (0.3)	1.000
Malignancy, *n* (%)	28 (10.2)	39 (13.4)	0.298
Previous stroke, *n* (%)	28 (10.2)	25 (8.6)	0.564
Prior MI, *n* (%)	14 (5.1)	13 (4.5)	0.844
LVEF, mean (%)	59.2 ± 13.2	60.1 ± 14.0	0.431
LVEF < 40%, *n* (%)	29 (10.6)	31 (10.6)	1.000
Killip Class ≥3, *n* (%)	27 (9.9)	27 (9.2)	0.886
Calcified lesion, *n* (%)	100 (36.5)	118 (40.4)	0.342
Bifurcation lesion, *n* (%)	21 (7.7)	22 (7.5)	1.000
Chronic total occlusion, *n* (%)	38 (13.9)	35 (12.0)	0.532
B2/C type lesion, *n* (%)	234 (85.4)	258 (88.4)	0.320
Clinical Frailty Scale, average	3.9 ± 1.4	4.0 ± 1.5	0.616
Clinical Frailty Scale ≥5	58 (21.1)	69 (23.6)	0.545

CR, complete revascularization; IR, incomplete revascularization; CKD, chronic kidney disease; COPD, chronic obstructive pulmonary disease, LVEF, left ventricular ejection fraction; MI, myocardial infarction; NSTE-ACS, Non-ST segment elevation acute coronary syndrome; PSM, propensity score matching.

**Table 4:** Clinical outcomes between IR and CR in NSTE-ACS in PSM cohort.

Clinical outcomes	Patient number, *n*	Event number, *n*	Event/patient number, %	Incidence per 1,000 person-years	Hazard ratio	95% confidence interval	*p*-value
All-cause mortality							
IR	274	37	13.5	49.5	1.00	[Reference]	
CR	292	16	5.5	18.9	0.39	0.22–0.70	0.002
CV mortality							
IR	274	18	6.6	24.1	1.00	[Reference]	
CR	292	6	2.1	7.1	0.30	0.12–0.75	0.010
Non-CV mortality							
IR	274	19	6.9	25.4	1.00	[Reference]	
CR	292	10	3.4	11.8	0.47	0.22–1.01	0.053
Non-fatal MI							
IR	274	6	2.2	7.9	1.00	[Reference]	–
CR	292	6	2.1	7.1	0.90	0.29–2.80	0.861
Any revascularization							
IR	274	29	10.6	38.6	1.00	[Reference]	–
CR	292	32	11.0	37.8	0.97	0.59–1.60	0.898
MACEs							
IR	274	54	19.7	76.1	1.00	[Reference]	–
CR	292	44	15.1	57.6	0.78	0.53–1.16	0.224

CR, complete revascularization; IR, incomplete revascularization; MACEs, major adverse cardiovascular events; MI, myocardial infarction.

## 4. Discussion

In this real-world propensity score matching cohort of elderly patients with ACS and MVD, we found that CR was associated with a lower risk of MACEs, mainly driven by lower all-cause mortality compared with IR after 3 years of follow up. There were no significant differences regarding non-fatal myocardial infarction or any revascularization. The trend of a better reduction in the risk of MACEs in the CR group than in the IR group was consistent across all pre-specified subgroups, but there was a greater benefit in the patients with an STE-ACS presentation.

The lower risk of MACEs with invasive treatment than with conservative treatment has been demonstrated in randomized studies of elderly patients with ACS (9). Though some observational studies have investigated the revascularization strategies in elderly ACS patients (5, 10–14), the randomized controlled trial addressing this issue is still ongoing (15). Since the COMPLETE (Complete vs. Culprit-Only Revascularization Strategies to Treat Multivessel Disease after Early PC) study demonstrated the superior outcome of complete revascularization (2), this strategy has been the standard of treatment in patients with STE-ACS and is recommended in current guidelines (1). These benefits were consistent, irrespective of patient age or lesion complexity (2). However, the COMPLETE study did not include elderly patients and the lesion complexity was relatively low (Mean SYNTAX score 16). Whether the benefit of complete revascularization can be generalizable to elderly patients remains debatable. Some observational studies have focused on revascularization strategies in elderly patients with STE-ACS, but the results are conflicting (5, 13, 16, 17). In summary, the studies enrolled relatively large population of elderly STE-ACS patients did show mortality benefit of CR (16, 17), and those studies with smaller populations did not demonstrate significant differences (5, 13). Although a moderate number of elderly patients presented with STE-ACS (*n* = 383) in the present study, the benefit of CR compared to IR in this subgroup was significant and the result is very similar to the findings of previous large registries (16, 17). Overall, patients in the CR group had significantly better outcomes at 3 years and the result is particularly significant in the STE-ACS subgroup (HR = 0.51, 95% CI: 0.31–0.85, *p* = 0.01).

In contrary to the STE-ACS setting, NSTE-ACS does not have much focus on revascularization strategies. Patients with NSTE-ACS could have a higher complexity of coronary anatomy and a higher proportion of elderly patients (11). The SMILE (Impact of Different Treatment in Multivessel Non-ST Elevation Myocardial Infarction Patients: One Stage Versus Multi-Staged Percutaneous Coronary Intervention) trial is the only randomized study in this field but the study is to compare one stage PCI with multi-stage PCI rather than CR vs. IR (18). Although there is increasing observational studies published in this field (11, 19), it remains unclear whether coronary revascularization of the presumed culprit lesion only or complete revascularization in NSTE-ACS patients should be attempted in the current guideline (20). In one of the big observational study, Agra-Bermejo et al. compared IR with CR in 500 pairs of elderly patients with NSTE-ACS and MVD and found that those with CR had a 26% lower risk of all-cause mortality than those with IR. In our study, we enrolled a relatively moderate number of elderly patients with NSTE-ACS and MVD (*n* = 566) and focused on the comparison between CR and IR (Table 3). The rate of achieving CR was 48.7% in this study, which was like that reported in previous studies (5, 12, 13, 16). Our results are very similar to Dr. Agra-Bermejo's findings that CR could better reduce the risk of all-cause mortality than IR (HR = 0.39, 95% CI: 0.22–0.70, *p* = 0.002). The survival benefit of CR is mainly relay on the reduction of cardiac mortality rather than non-cardiac mortality. However, the benefits of CR on MACEs risk reduction in NSTE-ACS were not significant (HR = 0.78, 95% CI: 0.53–1.16, *p* = 0.224) and do not seem to be as great as those in STE-ACS. Further study is needed to confirm this finding. It is surprising that the CR group did not have a significant reduction in myocardial infarction or revascularization in this study, since major randomized trials including myocardial infarction populations of all ages have reported that CR had the best effect on repeat revascularization or re-infarction (2, 21, 22). It is possible that older patients have higher complexity of coronary anatomy and may cause more suboptimal results of intervention, thus repeat revascularization are comparable in both groups. Another explanation is that older patients may have a higher risk of death than those with myocardial infarction or repeat revascularization, and are less likely to undergo repeat revascularization if they have functional disabilities, renal disease, or atypical presentation (23). Similar findings have been reported in other registries (12, 16).

Current guidelines recommend staged PCI of significant non-infarct artery stenosis to reduce the risk of death or myocardial infarction in selected hemodynamically stable patients with STE-ACS and MVD (1, 20). This study (excluded shock patients) also supports the concept that CR should be performed in elderly ACS patients with stable hemodynamic condition, regardless of STE-ACS or NSTE-ACS clinical presentation. However, patients should be carefully selected because most trials included younger patients with less complex disease (2, 17, 21, 22, 24). Regarding lesion complexity in our study, 88% of the participants had type B2/C lesions, 33% had calcified lesion, and approximately 20% had bifurcation lesions or chronic total occlusion. We suggest that interventions for complex diseases should not be limited in elderly patients if the risk-benefit ratio could be carefully weighed by physicians. Trying to achieve CR may have a meaningful benefit on mortality in selected elderly ACS patients after a thoughtful evaluation.

This study has several limitations. First, because of the retrospective design, the study groups may have had inherent differences. Although we used propensity sore matching to balance differences associated with major characteristics at baseline, hidden bias may still have occurred. Second, procedure details, CR success rate, and acute complications, including bleeding, and acute kidney injury were not collected in this study, and therefore we could not address the safety of CR in elderly patients. However, there was only one case had severe complication due to puncture wound related internal bleeding in the CR group. The in-hospital mortality rate was higher in the IR group (3.8%) than in the CR group (1.2%).

## 5. Conclusion

This observational study demonstrated that CR in elderly patients (≥75 years) with ACS and MVD is associated with a lower incidence of MACEs, mainly driven by lower risk of all-cause mortality. The observed trend of a more pronounced reduction in the risk of MACEs in the CR group, compared to the IR group, was consistent across all pre-specified subgroups. Moreover, interaction analysis revealed a greater benefit of CR over IR in reducing MACEs specifically in elderly patients with STE-ACS. Additionally, when analyzing the clinical outcomes in patients with NSTE-ACS, CR significantly had a lower risk of cardiac mortality than IR after 3-year follow-up.

## Data Availability

The raw data supporting the conclusions of this article will be made available by the authors, without undue reservation.
